# Median nerve hamartoma: Findings on magnetic resonance imaging

**DOI:** 10.4103/0972-2327.44566

**Published:** 2008

**Authors:** G. Chand, V. Chowdhury, Sapna Singh

**Affiliations:** Department of Radiodiagnosis, Maulana Azad Medical College and Lok Nayak Hospital, New Delhi, India

A 47-year-old patient presented with long duration pain in the right wrist and hand, with difficulty in writing and fullness of volar aspect of the wrist. Physical examination revealed fullness in the volar aspect of the wrist. Tinel's sign, indicating nerve compression was positive. Motor and sensory examination was otherwise normal. Clinically, the power of the muscles, supplied by the median nerve, was normal. The nerve conduction velocity (NCV) of the motor fibers of the median nerve was within normal limits, but the velocity for sensory fibers in distal median nerve was decreased (36m/s). The findings were consistent with carpal tunnel syndrome and the patient was referred for magnetic resonance imaging (MRI) examination of the wrist, to rule out any compression on the median nerve.

The MRI study revealed the presence of a large fusiform mass in the volar aspect of the wrist, replacing the median nerve. (Figures 1 and 2) Multiple low signal intensity bundles were seen clustered together, surrounded by high signal intensity, giving the typical appearance of *‘cable wire’*. The lesion was seen causing mass effect on the flexor retinaculum. The interspersed high signal intensity between the bundles was hyperintense on both T1W and T2W and followed the signal intensity of subcutaneous fat on all sequences, suggesting fatty component.

**Figure 1:**
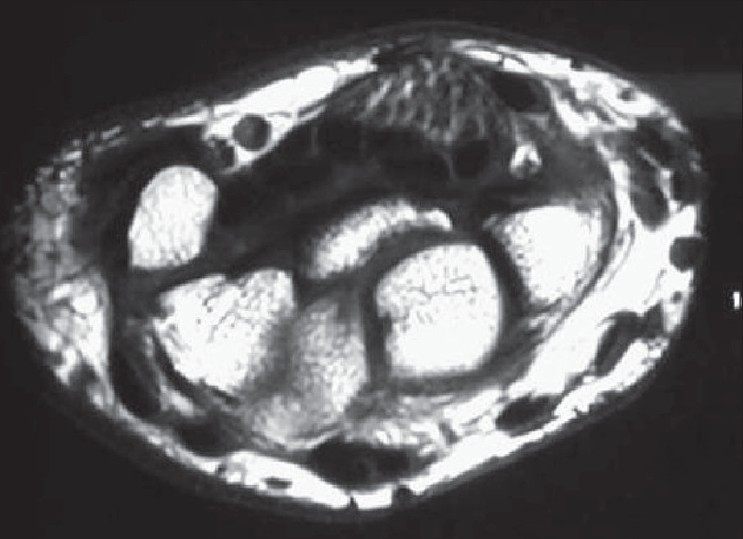
T1 weighted axial image of the wrist reveal bundle of hypointense circular structures beneath flexor retinaculum. The hypointense areas represent the neural bundles with perineural fibrosis. The nerve bundles are lying in a background of high signal intensity

**Figure 2:**
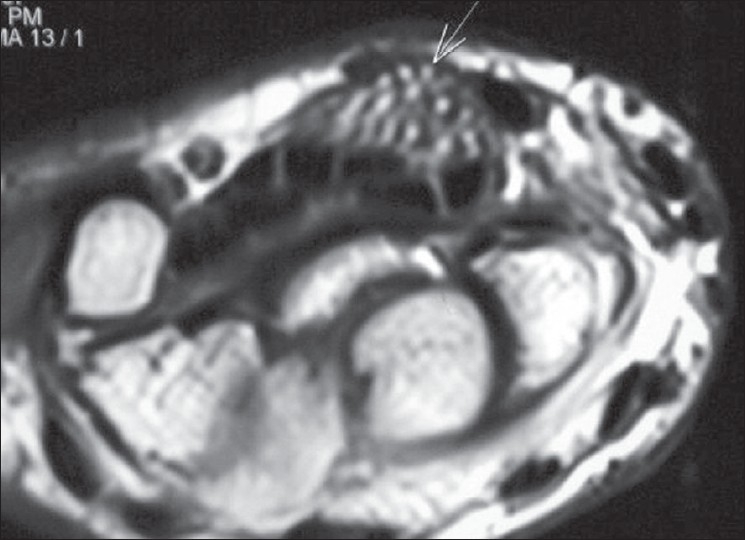
T2 weighted image at the same level shows of multiple nerve bundles (arrow) in the background of hyperintense signal which shows signal similar to subcutaneous fat suggesting fatty tissue. Flexor retinaculum is stretched above the lesion

The patient underwent surgery, where the flexor retinaculum was divided to decompress the carpal tunnel. However, resection of the tumor from the median nerve was not possible. Therefore, biopsy was taken and sent for histopathology, which confirmed the diagnosis. Postoperatively there was some improvement in the NCV from 36m/s to around 44m/s.

## Discussion

Fibrolipomatous hamartoma (FLH) of the median nerve has been considered a rare lesion, first reported in Western literature in 1953.[1] This lesion is still most frequently reported as affecting the median nerve and its branches.[2] FLH is an abnormal growth of the fibroadipose tissue of the nerve sheath, characterized by benign proliferation of epineural and perineural fibrous and mature adipose tissues, resulting in fusiform enlargement of the nerve. There is massive epineural and perineural fibrosis surrounding and compressing individual nerve bundles.[3]

Approximately, 80% of these lesions originate in the distribution of the median nerve.[2] Other reported sites of involvement include the ulnar nerve, radial nerve, the foot, brachial plexus, and cranial nerves.[4] Patients most commonly present before the age of 30. The swelling can be asymptomatic or accompanied by pain; there may be local motor or sensory symptoms, and late compression neuropathy. Approximately, two-thirds of the cases of fibrolipomatous hamartoma have associated macrodactyly, referred to as macrodystrophia lipomatosa. A mass is usually present for several years before the patient presents.[2]

MRI is useful in the preoperative evaluation of the lesion. Due to its multiplanar capabilities, it shows the true extent of the lesion and helps in presurgical planning. The MR appearance is characteristic, reflecting the exact morphology of the lesion. The individual nerve fascicles and the surrounding fibrosis result in long cylindrical bands of low T1 and T2 weighted signal intensity. The hamartomatous fatty tissue surrounding the nerve bundles appear bright on all sequences and follows the signal intensity of subcutaneous fat on all sequences. The longitudinally oriented, cylindrical regions of signal void, approximately 3 mm in diameter, seen on all sequences are thought to represent the nerve fascicles and accompanying epineural and perineural fibrosis. The presence of fat excludes neurofibromatosis, schwannoma, ganglion cysts, tenosynovitis and vascular malformations from the differential diagnosis of median nerve masses. Lipoma can also be excluded from the differential diagnosis, because lipoma will not infiltrate between the nerve bundles of the median nerve as does the median nerve hamartoma.[5,6]

The importance of accurate diagnosis of median nerve hamartoma has clinical relevance. Radical excision of the volar wrist masses has resulted in inadvertent permanent neurological deficits and hand dysfunction. Accurate preoperative diagnosis by MRI, therefore, can be of value in surgical management, which is controversial.[7] The current treatment practice is to provide initial decompression by tunnel release. Dissection and microsurgical excision is usually reserved for those cases with progressive and disabling median nerve compromise, despite previous carpal tunnel release. However, the technique can result in permanent loss of motor and sensory dysfunction.[8–10]
